# Learning dynamical systems from data: An introduction to physics-guided deep learning

**DOI:** 10.1073/pnas.2311808121

**Published:** 2024-06-24

**Authors:** Rose Yu, Rui Wang

**Affiliations:** ^a^Department of Computer Science and Engineering, University of California, San Diego, CA 92093; ^b^Department of Electrical Engineering and Computer Science, Massachusetts Institute of Technology, Cambridge, MA 02139

**Keywords:** deep learning, dynamical system, AI for science

## Abstract

Modeling complex physical dynamics is a fundamental task in science and engineering. Traditional physics-based models are first-principled, explainable, and sample-efficient. However, they often rely on strong modeling assumptions and expensive numerical integration, requiring significant computational resources and domain expertise. While deep learning (DL) provides efficient alternatives for modeling complex dynamics, they require a large amount of labeled training data. Furthermore, its predictions may disobey the governing physical laws and are difficult to interpret. Physics-guided DL aims to integrate first-principled physical knowledge into data-driven methods. It has the best of both worlds and is well equipped to better solve scientific problems. Recently, this field has gained great progress and has drawn considerable interest across discipline Here, we introduce the framework of physics-guided DL with a special emphasis on learning dynamical systems. We describe the learning pipeline and categorize state-of-the-art methods under this framework. We also offer our perspectives on the open challenges and emerging opportunities.

The explosion of real-time sensing data from the physical world opens up new opportunities for data-driven decision-making. While deep learning (DL) has made major breakthroughs in computer vision for images (1) and in natural language processing for text (2), its ability to process and reason about real-time dynamics over a wide range of spatial and temporal scales is still limited. In many disciplines of science and engineering, a common subject of study is dynamical systems (3), which are systems that evolve over space and time. Examples include fluid mechanics (4), transportation (5), and neuroscience (6). With the emergence of massive data, our ability to efficiently and accurately learn dynamical systems and extract useful knowledge is becoming more and more critical in scientific discovery.

Traditionally, dynamical systems models are *physics-based*: coupled differential equations describing known physical laws are solved over space and time via numerical schemes. These models are derived from first principles, can guarantee conservation laws, and are easy to interpret. Yet, physics-based approaches require strong modeling assumptions, and manually crafted features, and are typically computationally expensive. It is hard to simplify the model while maintaining satisfactory accuracy. For real-world systems with unknown dynamics, the strong assumptions in physics-based approaches can easily break down.

DL, on the other hand, is purely *data-driven*: Statistical models representing massive data are used to make predictions about the real world. DL allows flexible modeling of complex, unknown phenomena with minimal assumptions. It significantly speeds up dynamical system modeling by directly predicting input–output mapping and bypassing numerical integration. Recent work has shown that DL can significantly accelerate scientific simulations relative to numerical solvers, from turbulence modeling to weather prediction (7–9). This has triggered great excitement at the intersection of DL and scientific fields, such as molecular dynamics (10), epidemiology (11), and material science (12).

However, DL lacks a framework of physical reasoning (13)—the ability to understand and reason about physical laws. Without explicit constraints, they are prone to make physically implausible predictions, violating the governing physical laws (7, 14). DL models are often difficult to explain, and their predictions are uninterpretable to domain experts. Additionally, DL models often struggle with out-of-distribution generalization: Models trained on one dataset cannot adapt properly to unseen data from different distributions, known as distribution (covariate) shift. For dynamics learning, the distribution shift occurs not only because the dynamics are non-stationary and nonlinear, but also due to the changes in system parameters, such as initial and boundary conditions (15).

It is evident that neither physics-based models nor DL alone can handle the complexity of learning dynamical systems from massive real-world data. There is a growing need to integrate physics-based approaches with DL models, leading to the field of *Physics-Guided DL*. It offers a set of tools to blend physical concepts such as differential equations and symmetry with deep neural networks. On the one hand, DL models offer great computational benefits over traditional numerical solvers. On the other hand, the physical constraints impose appropriate inductive biases on the DL models, leading to scientifically valid predictions, reduced sample complexity, and guaranteed improvement in generalization to unknown environments.

Fig. 1 provides an overview of this framework. On the left, we list a few pros and cons of learning-based methods with representative tools such as neural networks and graphical models. On the right, we list a few pros and cons of physics-based models and several key concepts, such as differential equations and symmetry. Physics-guided DL expands tools in DL, and integrates concepts from physics-based models, leading to hybrid methods that inherit the best of the two worlds.

**Fig. 1.**
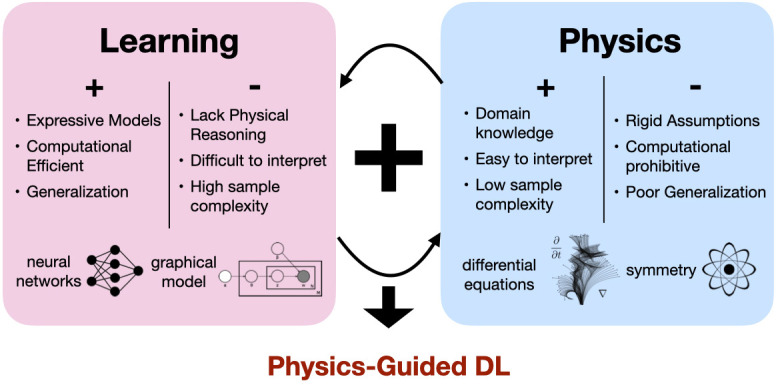
Overview of physics-guided DL. It combines complementary strengths of learning-based and physics-based methods, leading to efficient, expressive, generalizable models while pertaining to physical laws.

There is already a rich literature on physics-guided machine learning (4, 9, 16–18), but the focus on DL for dynamical systems is still nascent. This perspective paper introduces physics-guided DL to the general audience, assesses state-of-the-art approaches in the area, and offers new insights into its development. We begin with motivating scenarios in dynamical systems and then formalize the learning pipeline of physics-guided DL. We categorize existing approaches based on the strength of physics-based inductive bias in learning. We conclude with the open challenges in this field and discuss the emerging opportunities for future research.

## 1. Motivations in Learning Dynamical Systems

Learning dynamical systems from data efficiently and accurately has many practical values. This section describes several motivation scenarios where DL can play an important role in deepening our understanding of dynamical systems.

### A. Dynamical Systems Background.

Dynamical systems consist of variables that change over space and time and are ubiquitous in our daily lives. Formally, they are described with equations related to one or more unknown functions and their derivatives as below.

Definition 1: *[Dynamical System]*

Let the domain S be an open subset of $R^{d}$ and set an integer k≥1. Define the system state as $x:S\mapsto R^{m}$ where $x=(x^{1},...,x^{m})$. Then, an expression of the form:

[1] $$\begin{array}{c} F(D^{k}x\left(s\right),D^{k-1}x\left(s\right),...,Dx\left(s\right),x\left(s\right),s)=0 \end{array}$$

is called a $k^{\;\text{th}}$-order system of partial differential equation (or ordinary differential equation when d=1), where $F:R^{md^{k}}\times R^{md^{k-1}}\times ...\times R^{\mathrm{md}}\times R^{m}\times S\mapsto R^{m}$ and s∈S.

F models the dynamics of a m-dimensional system over a d-dimensional domain $s\in R^{d}$ and it can be either a linear or a non-linear operator. In general, one must specify appropriate boundary and initial conditions of Eq. **1** to ensure the existence of a solution. Since most dynamics evolve over time, s usually has a time dimension t. Without loss of generality, we will assume t to be the first dimension and use the shorthand $x_{t}\;\text{:=}\;x\left(t,\cdots \right)$ for the rest of the paper.

Learning the dynamical system is to identify the function F from data. It enables us to pinpoint the mathematical models underlying the system, improves our understanding of physical laws, makes accurate predictions, and helps to examine how the system behaves under different conditions.

### B. Accelerating Scientific Simulation.

When the underlying governing equation F in Eq. **1** is *known*, physics-based methods solve these equations via numerical schemes such as finite difference and finite element methods (19–23). However, when the system is high-dimensional and consists of many coupled equations, direct numerical simulation becomes prohibitively expensive. For non-linear dynamics that are common in atmospheric and ocean science, ensuring numerical stability in long-term prediction often requires fine-grained discretization, further increasing the computational cost. To make the computation feasible, existing scientific simulations have to compromise on the model accuracy with reduced order models or approximate solutions (24).

DL has demonstrated great success in the automation and acceleration of scientific simulation. With a one-time cost of training the model on massive data, it bypasses the expensive numerical integration steps and directly predicts the desired numerical quantities during inference (7, 25, 26). In other cases, DL is used to approximate the final solution of the differential equations. It acts as an efficient finite-difference solver through automatic differentiation (auto-diff), leading to the framework of physics-informed neural networks (PINNs) (27–30). In many applications, DL models are orders of magnitude faster than classic numerical simulation tools, including fluid simulation (8), weather and climate modeling (9), and molecular dynamics (10).

### C. Approximating Unknown Dynamics.

When F in Eq. **1** is *partially unknown*, we can use DL to fill in the missing terms in physical models. For example, for complex systems like jet engines, epidemics, or magnetic fusion devices, it is very difficult to fully characterize the underlying physics. Even though the fundamental principles of dynamics still hold in these systems, measurement noise, environment disturbance, and emergent physical phenomena introduce many unknown factors in the governing equations. Even when the governing equation is fully known, some terms in the equation may be too difficult to compute. Using DL to approximate those terms can also reduce computational costs.

With sufficient data and hidden units, (deep) neural networks are universal function approximators (31). Hence, DL acts as a convenient vehicle to extract the unknown dynamics from data. Here, the physics-based model is augmented with an additional DL component. The resulting hybrid model corrects the potential bias of the partially known physics model and provides a more accurate description of the dynamics, hence facilitating downstream modeling, prediction, and analysis. However, the drawback of such an augmented model is “interpretability.” Different from physics-based models, the black-box DL model does not offer explicit functional relationships for the physical quantities of interests. Examples of such models include stabilizing quadrocopter control (32), improving epidemic forecasting (33), designing better lake temperature models (34), and solving the closure problem in turbulence models (35).

### D. Discovering Governing Equations.

When F in Eq. **1** is *unknown*, and we prefer traditional physics-based models for explainability, DL can also be helpful in the discovery of the models. In general, the automatic discovery of governing equations is challenging. Real-world systems frequently involve many interdependent variables with non-linear relationships. The available data may be noisy or incomplete. For high-dimensional data, the curse of dimensionality makes it even harder to extract meaningful patterns and relationships. Despite these challenges, DL has demonstrated promises to automate the process of identifying complex, nonlinear dynamics models from data.

There are generally two types of approaches in equation discovery: symbolic regression (36, 37) and sparse regression (38, 39). Symbolic regression uses a search-based algorithm to assemble mathematical expressions and parameters while minimizing certain fitting errors. For example (40), extends symbolic regression to a high-dimensional setting by using a graph neural network to encourage sparse latent representations. Petersen et al. (41) employs a recurrent neural network (RNN) to emit a distribution over mathematical expressions and train the RNN model with reinforcement learning.

Sparse regression takes a regression approach to select from a large set of mathematical basis functions via regularization. For instance, Brunton et al. (38) and Schaeffer (42) discover ordinary differential equations by creating a dictionary of possible basis functions and selecting sparse, low-dimensional, and nonlinear models from the data. Lagergren et al. (43) and Rudy et al. (44) incorporated neural network modules to further augment the dictionary of basis functions for more complex dynamics. Martius and Lampert (45) and Sahoo et al. (39) presented a shallow neural network approach to identify concise equations from data. They replaced the activation functions with predefined basis functions, including identity and trigonometry functions, and used specially designed division units to model division relationships in the potential governing equations.

### E. Designing Generalizable Dynamics Models.

Both physics-based mechanistic models and black-box DL models can learn the underlying dynamics from data and make predictions. Traditional dynamics models often emphasize model fitness to existing data (5, 46, 47). A good dynamics model is one that can perfectly describe the behavior of the current measurements. It puts less emphasis on the model’s ability to predict well on unseen domains. The standard DL paradigm of training–validation–testing is often missing in the traditional dynamics modeling literature. Therefore, for systems with even slightly different initial, boundary conditions, and system dynamics parameters, a different model has to be rebuilt to ensure better fitting.

One advantage of DL dynamics models over the traditional dynamical models is their strong emphasis on *generalization*, i.e., the ability to perform well in the data regimes beyond the training dataset. DL models are fundamentally statistical. Hence, they have the ability to generalize and make accurate predictions in unseen scenarios, especially in-distribution generalization. In dynamical systems, statistical approaches to extract mathematical models from observed data have roots in system identification (ID) (48, 49). But DL further generalizes system ID with more expressive architecture, efficient computation, and minimal modeling assumptions. DL models typically require pre-specified model architectures, initialization schemes, and optimizers. But there are also advances in DL that automatically discover DL models from data using neural architectural search (50).

## 2. The Learning Pipeline

DL pipeline commonly contains three main ingredients: 1) data, 2) model, and 3) learning objective. In this section, we first describe standard data preparation steps in dynamical system learning. Then, we will discuss the empirical observations for DL model architectures. The learning objective depends on the task. We will use a few examples to illustrate the design of learning objectives.

### A. Data Preparation.

Learning dynamical systems is to search for a model F that can accurately describe the behavior of the physical process given the measurement data. Formally, given a set of data sequences (samples) from an unknown dynamical system F as D, a key assumption in machine learning is that the samples in the dataset D are independently identically distributed (i.i.d.). However, due to the spatiotemporal dependencies, the data generated by dynamical systems usually do not satisfy this assumption.

One way to resolve this conflict is to randomize the parameters of the systems including the initial conditions and the coefficients. For each parameter configuration, we generate an independent sequence of states x(s) as a sample. However, in practice, we may not have direct control of the system parameters, such as the sea surface temperature in the ocean. Instead, what we observe is a long sequence of states from the same system. In this case, we need to assume that states only have short-term dependencies. Within a pre-defined window, the states are interdependent. Beyond the window, the sequences can be treated as independent.

Under this assumption, we can use a *rolling-window* approach to construct a dataset of samples. Suppose that we have measured a long sequence of states from a dynamical system $\left(x_{0},\cdots ,x_{j},\cdots ,x_{T}\right)$. Let the window size be W, the rolling window moves along the time axis and extracts sub-sequences of length W as samples. To reduce the overlap between two adjacent sub-sequences, we introduce the time gap between two samples as stride S. If the stride is large, then two samples would be less dependent. In this way, we can obtain the dataset with a new time index $D={\left\{\left(x_{i*S},x_{i*S+1},...,x_{i*S+W-1}\right)\right\}}_{i=0}^{\left\lfloor (T-W)/S\right\rfloor }$ as sub-sequences extracted from the original sequence. Here, the notation ⌊x⌋ gives the largest integer that is smaller than x.

### B. Model Selection.

Designing a DL model often requires careful selection of layers (building blocks of DL), activations (nonlinearity), depth (number of layers), width (number of hidden units in a layer), and regularization (dropout, batch norm, etc.). Despite the progress in DL optimization and generalization theory (51, 52), there exist very limited practical guidelines for model selection in DL. Therefore, the design of the DL model architecture still largely relies on engineering intuition and trial and error.

For low-dimensional time series data from dynamical systems, the traditional view is that it can be better modeled by a Recurrent Neural Network (RNN) and its variants such as Long Short Term Memory (LSTM) (53) and Gated Recurrent Unit (GRU) (54). The common belief is that RNNs are discrete-time dynamical systems with trainable weights; they are more suitable for learning dynamical systems. Yet, RNNs suffer from vanishing gradient problems and are difficult to train. Its sequential processing nature also limits its applicability to large datasets.

Transformers (2), being particularly successful in vision and language applications, have also been extended to the time series domain (55). Transformers use non-autoregressive encoding, and hence are more compatible with modern DL hardware with massively parallel processing power. However, recent benchmark experiments in time series forecasting have revealed surprising results of fully connected networks (56), even outperforming transformers.

For high-dimension spatiotemporal dynamics, Convolutional LSTM (ConvLSTM) (57) and PredRNN (58) were proposed to model spatial and temporal dependencies. However, since RNN/LSTM uses sequential processing rather than parallel processing, they often suffer from scalability issues. Pure convolutional architectures such as ResNet and UNet are shown to be more efficient baselines for spatiotemporal modeling (7, 59). Transformers have also been generalized to spatiotemporal prediction problems as well (60). Unfortunately, even now, there is no widely accepted “foundational model” for learning dynamical systems.

### C. Learning Objective.

We explain the design of the learning objective for some examples of tasks in learning dynamical systems, namely 1) solving differential equations, 2) dynamic forecasting, and 3) discovering governing equations.

#### C.1. Solving differential equations.

Solving the differential equation in Eq. **1** means finding a functional form x(s)=ψ(s) that satisfies the differential equation. For complex equations, instead of relying on expensive numerical integration, we can directly approximate its solution of ψ(s) with a deep neural network. In particular, given a dataset D of size N, denote the DL model as ${\hat{f}}_{\theta }$ with trainable weights θ, we want to learn a model that fits well with the data and also satisfies the governing equations (27, 61). We can design the following learning objective to jointly optimize both,

[2] $$\begin{array}{c} \;\text{min}\underset{\theta }{\;}\frac{1}{N}\sum_{x(s)\in D}L\left(x\left(s\right),{\hat{f}}_{\theta }\left(s\right)\right)+\lambda_{\theta }R\left(F\left(D^{k}{\hat{f}}_{\theta }\left(s\right),\cdots ,s\right)\right). \end{array}$$

The first term L(·,·) in Eq. **2** quantifies the loss between the DL predictions and the training data points. The second term R(·) is a regularization on the governing equation. The constant $\lambda_{\theta }$ is a parameter that balances the prediction error and regularization. This is also the key idea of PINNs (18) that are particularly effective in solving inverse problems. A common choice for the loss is the mean square error and the regularization can be the $L_{2}$ norm. Recent research in (62) has found that $L_{2}$ loss is not suitable for training PINNs on a certain class of high-dimensional nonlinear PDEs.

#### C.2. Dynamics forecasting.

When the governing equation is unknown, dynamics forecasting learns from data by directly predicting the input–output state mapping (9, 63, 64). Given the dataset D, we can formulate the problem of dynamics forecasting for a m-dimensional system as learning a model parameterized by θ, ${\hat{f}}_{\theta }:R^{m\times k}\mapsto R^{m\times q}$ that maps historic states to future states of the dynamical system:

[3] $$\begin{array}{c} {\hat{f}}_{\theta }:\left(x_{t-k+1},\cdots ,x_{t}\right)⟶\left(x_{t+1},\cdots ,x_{t+q}\right), \end{array}$$

where k is the input length and q is the output length. In the nomenclature of forecasting, k is the time lag and q is the forecasting horizon. The forecaster model ${\hat{f}}_{\theta }$ essentially approximates the forward dynamics of the system.

Consider a simplified single-step forecasting setting where q=1, then the learning objective is as follows:

[4] $$\begin{array}{c} \;\text{min}\underset{\theta }{\;}\frac{1}{N}\sum_{x_{1:t+1}\in D}L\left(x_{t+1},{\hat{f}}_{\theta }\left(x_{t:t-k+1}\right)\right). \end{array}$$

Here, we use $x_{t:t-k+1}$ as a shorthand for $\left(x_{t-k+1},\cdots ,x_{t}\right)$. The goal is to minimize the error between the ground truth and the predicted state. When q>1, we face a more challenging scenario of multi-step forecasting. A straightforward approach is to decompose the output sequence into q single-steps and minimize the total error:

[5] $$\begin{array}{c} \;\text{min}\underset{\theta }{\;}\frac{1}{N}\sum_{x_{1:t+1}\in D}\sum_{j=0}^{q-1}L\left(x_{t+j+1},{\hat{f}}_{\theta }\left(x_{t+j:t+j-k+1}\right)\right). \end{array}$$

However, the target states ${\left\{x_{t+j+1}\right\}}_{j=0}^{q}$ are only available during training. At inference time, one has to use the predicted state instead of the target state as input to the trained model and auto-regressively generate multiple-step forecasts. Hence, the training and testing data for the model become different, leading to distribution shifts known in structured prediction. There exist many ways to alleviate this issue through data augmentation (65), scheduled sampling (66), and adversarial training (67). However, stable long-term forecasting remains an open challenge.

#### C.3. Discovering governing equations.

Given the observed data, we are interested in determining the underlying mathematical equations that govern the dynamics. At a high level, we first construct a set of candidate mathematical expressions as building blocks and then search for the best combination of these building blocks that fits the data. More specifically, define $\Phi \left(x\left(s\right),s\right)=\left[\phi_{1}\left(x,s\right),\ldots ,\phi_{p}\left(x,s\right)\right]$ as a set of basis functions, such as polynomials and trigonometric functions. Assume a DL model ${\hat{f}}_{\theta }$ combines these basis to form the governing equation. The problem of discovering governing equations can be formulated as follows:

[6] $$\begin{array}{c} \;\text{min}\underset{\theta }{\;}\frac{1}{N}\sum_{x(s)\in D}L({\hat{f}}_{\theta }\left(\Phi \left(x\left(s\right),s\right),0\right)+\lambda_{\theta }R\left(\theta \right), \end{array}$$

where $\lambda_{\theta }$ is the regularization constant and R(θ) is commonly $L_{1}$ norm. The first term measures the fitness of data and the second term encourages sparsity in coefficients.

## 3. Physics-Guided DL

**Fig. 2.**
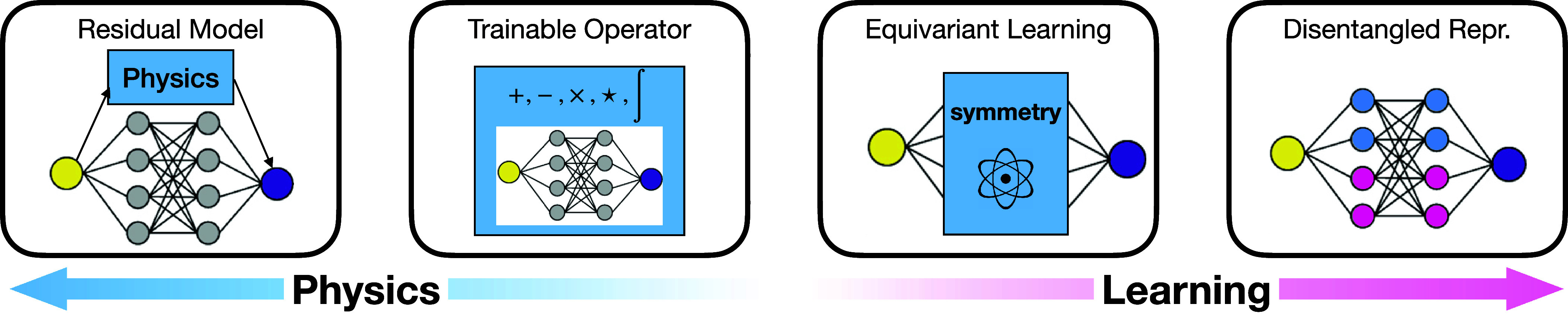
Four approaches in physics-guided DL with stronger physics toward the left and more dependency on learning from data toward the right.

From data to models to learning objectives, there are various ways in which physics can be an inductive bias to guide learning. In this section, we categorize the state-of-the-art approaches into four groups with increasing dependencies on the data: residual model, trainable operator, equivariant learning, and disentangled representation, shown in Fig. 2.

### A. Residual Model.

When we have measurement data and partially known physics, one way to integrate physics and DL is to combine them together. As an example, assume the true dynamics F can be written in an additive form F=g+r, where g represents the known physics model and r is the residual term that encompasses measurement noise, possible model misspecification, and unknown physics. We can use traditional methods to solve the part in g and a DL model to approximate the dynamics of r (68, 69).

In general, a residual model uses DL to learn the difference between physics-based models and observed data. Here, we use the word “residual” as a general concept to represent the potential bias or errors of a physics-based model. We use the simple additive form of the residual as an example, but the method also applies to other forms of residuals. Formally, given observed data D, let the DL model be ${\hat{f}}_{\theta }$ with parameters θ. If the physics model is fully differentiable, then we can jointly minimize the following objective:

[7] $$\begin{array}{c} \;\text{min}\underset{\theta }{\;}\sum_{x(s)\in D}L\left(x\left(s\right),g\left(s\right)+{\hat{f}}_{\theta }\left(s\right)\right). \end{array}$$

Here, the physics-based model is part of the learning objective and is computed using automatic differentiation.

When the physics-based model is not differentiable, we can opt for a two-stage training approach. We first simulate the data with the physics-based model. Then, we calculate the difference between the simulated data and the observed data as $D^{\prime }=\left\{x\left(s\right)-g\left(s\right)|x\in D\right\}$. Treating the residual $D^{\prime }$ as the new training data, we can then directly optimize the DL model to predict the residuals

[8] $$\begin{array}{c} \;\text{min}\underset{\theta }{\;}\sum_{r\left(s\right)\in D^{\prime }}L\left(r\left(s\right),{\hat{f}}_{\theta }\left(s\right)\right). \end{array}$$

Care needs to be taken when using DL for residual modeling. In certain scenarios, the governing dynamics may need to be stable (32), or the states need to be non-negative (11). Without imposing additional constraints on the residual model, the learned DL model may become too flexible, leading to undesirable behavior.

### B. Trainable Operator.

In mathematics, an operator is a mapping from a space of functions to another space of functions. Some canonical examples of operators include linear operators, differential operators, and Fourier operators. Operators are instrumental in dynamical system modeling. However, traditional physics-based models rely on hand-designed operator functions, which are error-prone and labor-intensive. The trainable operator addresses this issue by parametrizing the operator with trainable neural networks.

Generally speaking, trainable operators or neural operators define an emerging class of architectures that enable learning in the space of functions (70–73). Let an operator be F, that maps between functions F:U→V where U and V are both separable Banach spaces of real-valued functions. For example, a Laplacian operator takes the integral form $F\left\{f\right\}\left(s\right)=\int_{0}^{\infty }e^{-st}f\left(t\right)dt$ for a given function f(t)∈U. The operator is parameterized by a given function $e^{-st}$. We can assign trainable parameters θ to the operator as $F_{\theta }$ and learn the values of these parameters from the data. After training, the learned operator still maintains the same mathematical properties but is more flexible, and can potentially be a better fit for the prediction task as well.

For data in a finite domain, trainable operators have already been used for the design of convolutional neural networks (CNNs). For example (7), introduces trainable convolutional operators in Computational Fluid Dynamics (CFD). Specifically, the Hybrid RANS-LES Coupling technique (74) computes the spatial and temporal averaging of the turbulent flow x(s,t) using a convolutional operator:

[9] $$\begin{array}{cc} x*(s,t) & =S*x=\sum_{\xi }S\left(s|\xi \right)x\left(\xi ,t\right), \end{array}$$

[10] $$\begin{array}{cc} \overset{¯}{x}(s,t) & =T*x*=\frac{1}{n}\sum_{\tau =t-n}^{t}T\left(\tau \right)x*\left(s,\tau \right). \end{array}$$

Here, the state x of the turbulence represents the pressure, velocity field and (s,t) are the discretized space and time coordinates. By parameterizing the operator S and T with CNNs, we can obtain a deep surrogate model for turbulent flow prediction that satisfies the physics of fluid flow.

Besides the finite-dimensional domain, trainable operators also have the ability to handle infinite-dimensional functions. Given a domain S and an input function f, according to Li et al. (75), a more general form of the trainable operator is as follows:

[11] $$\begin{array}{c} F\left\{f\right\}\left(s\right)=\int_{S}K\left(s,t,\Pi \left(s\right),\Pi \left(t\right)\right)f\left(t\right)d\nu \left(t\right)\;\forall t\in S, \end{array}$$

where K is a parameterized convolutional kernel, Π is a mapping that can depend on the input function f, and ν can be the Lebesgue measure on $R^{d}$ for a d-dimensional domain. Here, both the s and t can take any continuous value.

Given finite data points sampled at arbitrary positions of the domain, trainable operators learn a representation of infinite-dimensional function space with parameterized kernels. During inference, the model can also be queried continuously anywhere in the prediction domain. Such discretization invariant models resolve the issue in finite dimensions when the data have different resolutions and require different DL models for each resolution.

Trainable operators have already demonstrated success across a wide range of applications including fluid mechanics and climate modeling (76, 77). For example, Li et al. (75) proposed a graph neural operator that learns the mapping between function spaces and is invariant to different grid sizes. It used the message-passing graph network to learn Green’s function from the data, and then, the learned Green’s function can be used to compute the final solution of PDEs. Li et al. (76) further extended it to the Fourier Neural Operator by replacing the kernel integral operator with a convolution operator defined in Fourier space, which is much more efficient.

### C. Equivariant Learning.

In physics, Noether’s theorem gives a correspondence between conserved quantities and groups of symmetries. For example, time invariance corresponds to energy conservation. Space translation symmetry gives momentum conservation. Therefore, by incorporating symmetry in DL, we naturally satisfy conservation laws.

Equivariant neural networks (ENNs) use symmetry as an inductive bias to design deep neural network architectures. A deep neural network can be viewed as the composition of functions. The fundamental design principle of ENNs is that the composition of equivariant functions is equivariant. Formally, a function f:X→Y may be described as respecting the symmetry coming from a group G using the notion of equivariance. Assume a group representation $\rho_{\;\text{in}}$ of G acts on X and $\rho_{\;\text{out}}$ acts on Y. We say a function f is *G-equivariant* if

[12] $$\begin{array}{c} f\left(\rho_{\;\text{in}}\left(g\right)\left(x\right)\right)=\rho_{\;\text{out}}\left(g\right)f\left(x\right), \end{array}$$

for all x∈X and g∈G. The function f is *G-invariant* if $f(\rho_{\;\text{in}}\left(g\right)\left(x\right))=f\left(x\right)$ for all x∈X and g∈G. This is a special case of equivariance for the case $\rho_{\mathrm{out}}\left(g\right)=1$.

Therefore, if the linear layers in a deep neural network and activation functions are equivariant, then the entire neural network will be equivariant. A well-known example of this principle is CNNs for image classification, where each convolution is equivariant with respect to translations. This, when combined with pooling, ensures that the predicted class label will be invariant to translations.

**Fig. 3.**
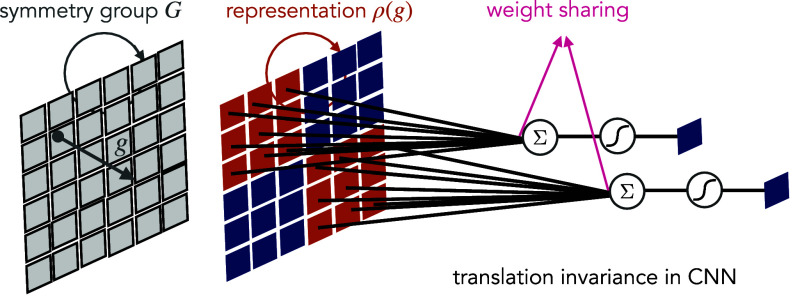
Illustration of symmetry in DL: the input data (image) is defined on the domain (grid Ω), whose symmetry (translation group g) acts on the input through the group representation ρ(g) (shift operator). Enforcing the underlying neural network functions f (e.g., image classifier) to satisfy translation invariance.

Equivariant linear layers can be produced by *weight-sharing*. For example, in a convolution layer, as we translate the input, the feature maps are also translated. CNN achieves translation invariance by sharing the weights in the block diagonal elements in the kernel, as shown in Fig. 3. If the output does not transform at all, the function is invariant to the action of g. Such a weight-sharing scheme also highlights the role of symmetry in reducing the number of parameters in the model, which leads to computational benefits.

In general, Cohen et al. (78) proved that a convolutional network is G-equivariant and $R^{2}$-translation equivariant if and only if

[13] $$\begin{array}{c} K\left(gv\right)=\rho_{\;\text{out}}\left(g\right)K\left(v\right)\rho_{\;\text{in}}\left(g^{-1}\right)\;\;\text{for all}\;g\in G,\;v\in R^{2}, \end{array}$$

where $K\in R^{2}\to R^{c_{\mathrm{in}}\times c_{\mathrm{out}}}$ is a convolution kernel. $\rho_{\mathrm{in}}:G\to \mathrm{GL}\left(R^{c_{\mathrm{in}}}\right)$ and $\rho_{\mathrm{out}}:G\to \mathrm{GL}\left(R^{c_{\mathrm{out}}}\right)$ are input and output group representations, yielding matrices that act on the vector space of $R^{c_{\mathrm{in}}}$ and $R^{c_{\mathrm{out}}}$. Such a group convolution structure is a sufficient and necessary condition for a CNN to be equivariant (79). This theory gives a general guideline for designing neural networks for data with non-trivial symmetries. Essentially, given a group g, we need to design the appropriate weight-sharing or weight-tying schemes in the kernel K(v) of a network layer.

For dynamical systems, symmetry is defined in terms of their solutions. Consider a system of differential operators F acting on x. Denote the set of solutions Sol(F)⊆x. We say G is a symmetry group of the dynamical system F if G preserves Sol(F). That is, if ψ is a solution of D, then for all g∈G, g(ψ) is also a solution. For example, Navier–Stokes equations have time translation and scaling symmetry. We can design corresponding ENNs that satisfy these symmetries, leading to improved generalization in deep dynamics forecasting (64).

ENNs for dynamical systems have been successfully used to predict turbulent flow (64), forecast trajectories (80), learn robotic manipulation (81), and generate novel molecules (82). Apart from perfect symmetry, extensions of ENNs that account for imperfect dynamics and uncertainty in the data have also been studied. For example, Wang et al. (83) studied a family of approximate ENNs with relaxed weight-sharing that can handle symmetry-breaking in the dynamics. Sun et al. (84) extended the notion of probabilistic symmetry (85) to capture the uncertainty during the real-world decision-making process.

### D. Disentangled Representation.

DL models can learn rich representations from data. However, without explicit constraints, the learned representations may not be physically meaningful or easily interpretable. Disentangled representation is a class of techniques in DL that either uses statistical criteria or physical principles to separate the underlying structures in the learned latent representations. The word “disentanglement” comes from quantum mechanics and refers to the separation of variations or removal of dependencies (86). Disentangled representation is a weaker form of inductive bias as the physics constraint is only imposed implicitly in the latent space rather than explicitly on the predictions.

While there is no widely accepted definition of disentanglement in the community, many measure the level of disentanglement by quantifying the statistical relationships of the learned structures. Most of these methods are based on the framework of Variational Autoencoder (VAE) (87) where a parametrized posterior over latent variables q(z|x) is learned. For example, Kim and Mnih (88) augment VAE with a total correlation term $\mathrm{KL}(q\left(z\right)||\sum_{j=1}^{n}q\left(z_{j}\right))$ on the latent representations. This directly encourages independence in the distribution of n latent variables $\left\{z_{j}\right\}$. Higgins et al. (89) defines disentangling through symmetry transformation where symmetries are decomposed into n sub-groups $G=G_{1}\times \cdots \times G_{n}$. The space of representations is also factorized as $Z=Z_{1}\times \cdots \times Z_{n}$. Disentangled representation means that the representation $Z_{i}$ is only affected by $G_{i}$ but not by the others.

Similar notions of disentanglement have also been generalized to physical systems. For example, by leveraging the functional separation of variables in PDEs, Donà et al. (90) propose a model for spatiotemporal disentanglement. They combine a prediction objective and penalties to decompose the solution into a spatial PDE and temporal ODE. Quessard et al. (91) use the disentanglement definition of Higgins et al. (89). They introduce a disentanglement regularization such that each transformation acts on a minimum of dimensions of the latent space. Disentangled representation has also been used for meta-learning (92) that can generalize across heterogeneous domains of dynamics. In most cases, the disentangled representation encourages simple, low-dimensional structures in the latent space that explain the physical phenomena of interest.

## 4. Challenges and Future Directions

We have introduced the background, motivation, formulation, and methodologies in physics-guided DL, applied to dynamical systems. There are still many challenges in the field that present emerging opportunities for future research.

### A. Generalization under Distribution Shift.

Current DL models for complex dynamics are still limited by their ability to generalize to new domains. In machine learning, generalization implies statistical robustness against distribution shifts. For dynamical systems, distribution shift can occur not only in the data distribution but also in system parameters (93). For example, in turbulence modeling, DL models trained with fixed boundaries and initial conditions can fail to generalize to fluid flows with different characteristics. However, the distribution of the states may still be similar.

Residual models use physics equations to improve generalization. Equivariant networks enable the models to generalize within the group transformation. But most DL models can only generalize within the interpolated range of parameters, but cannot *extrapolate* beyond the parameter range in the training set. Rackauckas et al. (94) build universal differential equations (UDEs) that use a known physics-based model form with missing terms defined by a DL model. With the correctly discovered governing equations, their UDEs can accurately extrapolate beyond the original data for low-dimensional time series. Despite much work in handling distribution shifts in DL, tailored solutions for modeling high-dimensional physical dynamics are still in their infancy.

### B. Stability and Robustness.

A different notion of robustness in dynamical system modeling is stability. It implies the ability to generate long-term forecasts and long-range predictions. As explained in Section C.2, long-term multi-step forecasting is prone to error accumulation. Non-linear chaotic dynamics are extremely sensitive to small perturbations in the initial conditions. Even with traditional numerical models, having stability guarantees often requires very strong assumptions on the input data distribution and underlying system dynamics. Since the theory of non-linear dynamics is not well understood, when combined with DL, very little guarantee exists to certify the stability of the learned model.

Control theory offers useful tools to improve the stability and robustness of DL predictions. For example, Miller and Hardt (95) analyze the stability of recurrent models by upper-bounding the Lipschitz constant of the functions. Zico Kolter and Manek (96) propose to jointly learn a dynamics model and a Lyapunov function that guarantees the stability of the dynamics under the learned Lyapunov function. Data augmentation (97) and data normalization (26) techniques also make the models more robust to input perturbations and distribution shifts. However, many of these models require a large amount of simulation data. In practice, obtaining real-world data can be expensive, which presents a significant challenge in improving the long-term stability and robustness of predictions.

### C. Uncertainty Quantification.

Another challenge in learning dynamical systems is uncertainty quantification (UQ). Epistemic uncertainties arise due to noise in the training data, the choice of the model, and the variability of its predictions due to stochastic optimization algorithms. Aleatoric (chaotic) uncertainty comes from the choice of predictive variables being an incomplete representation of the underlying system. For dynamical systems, sensitivity analysis (98) is often used to quantify error propagation using Monte Carlo methods.

DL models are poor at providing uncertainty estimates and tend to produce overconfident predictions. It is also difficult to completely separate out aleatoric and epistemic uncertainties. The strong spatio-temporal dependencies, out-of-distribution shifts, and a large number of dimensions in dynamical systems pose further challenges to UQ in DL. There are roughly two types of UQ methods. One leverages frequentist thinking and focuses on robustness. Perturbations are made to the inference procedure in initialization (99), neural network weights (100), and datasets (101). The other type is Bayesian, which aims to model posterior beliefs of network parameters given the data (87). Both approaches can be cast under a unified framework via statistical decision theory (102). For dynamical systems, UQ requires carefully designed evaluation metrics, calibration techniques (103), and the ability to handle multifidelity uncertainty (104).

### D. Non-Euclidean Geometry.

Many of the dynamical systems studied in DL follow Euclidean geometry on a “flat” surface. Dynamical systems in non-Euclidean space such as a sphere or hyperbola bring many new challenges. The primary difficulty lies in the renewed notions of points, angles, and distance in curved spaces. There is a lack of a global vector space structure and a canonical ordering of neighbors. Geometric DL (105) generalizes DL models to non-Euclidean domains. However, most of the existing work is limited to static data, without much consideration of dynamics. Therefore, developing physics-guided DL models for non-Euclidean dynamics is a major challenge. Here, we must jointly account for dynamical systems fundamentals, and geometric concepts, such as notions of distance, curvature, and parallel transport. For example, when modeling the dynamics of oceans on Earth, we need to encode the gauge equivariance (106) in the design of neural nets, as there is no canonical coordinate system on a sphere.

### E. Explainability and Causality.

A fundamental pursuit in science is to identify causal relationships. These relationships also provide mechanistic explanations of various outcomes in our world. For dynamical systems, we may ask which variables influence other variables through intermediates or how causes lead to effects. Grounded in probabilistic graphical models, causal models allow us to ask both predictive and counterfactual queries. Causality can also model distribution shifts by encoding both observational and interventional distributions. Classic causal discovery framework involves conducting randomized experiments (107). However, randomized experiments are not always possible; this requires us to discover causality from observational (108). Under certain assumptions, we can identify the underlying causal model using constrained algorithms to test for independence or score-based approaches to search for structures (109). However, these algorithms still struggle in big data settings with a large number of dimensions. Physical laws are causal laws. It is an interesting question of how to impose such inductive biases in physics-guided DL as causal priors. Furthermore, identifying causal variables from high-dimensional data and disentangling multiple treatments from dynamic data are also important challenges.

### F. Theoretical Guarantees.

The majority of literature on DL for dynamical systems focuses on methodological and algorithmic advances. To develop a fundamental understanding of these methods, we need to establish principled theory toward a deeper understanding of their properties in approximation, optimization, and generalization. For example, classic statistical learning theory for generalization assumes that data are i.i.d. samples from some unknown distribution, which is easily violated by dynamic data. Kuznetsov and Mohri (110) provided the generalization guarantees based on discrepancies for time series forecasting. Wang et al. (111) took the first step to derive generalization bounds for equivariant models and data augmentation for dynamics forecasting. However, to better understand the performance of DL in learning dynamics, we need to consider properties of the dynamics, such as the governing equations and Lyapunov exponents. The functional approximation theorems (31) can also be relevant in quantifying the expressive power of various models (112). Another emerging area of study is the interplay between the optimization dynamics in DL and the dynamics of the underlying data (113). With a deeper understanding, these theoretical studies can inspire new developments in model and algorithm design to learn complex dynamics.

## 5. Conclusion

With the emergence of real-time data and rapid growth in computation, DL is posed to bring new opportunities to science and engineering. Toward the long-term goal of AI scientists, it is critical that we integrate domain knowledge in a systematic manner when developing data-driven models. This perspective paper provides a gentle introduction to the framework of physics-guided DL for learning dynamical systems. The continued development in this field will play an essential role in addressing significant dynamics modeling problems, developing trustworthy AI models, and eventually transforming the future of scientific discovery.

## Data Availability

There are no data underlying this work.
